# Mid-Level Healthcare Personnel Training: An Evaluation of the Revised, Nationally-Standardized, Pre-Service Curriculum for Clinical Officers in Mozambique

**DOI:** 10.1371/journal.pone.0102588

**Published:** 2014-07-28

**Authors:** Caryl Feldacker, Sergio Chicumbe, Martinho Dgedge, Gerito Augusto, Freide Cesar, Molly Robertson, Francisco Mbofana, Gabrielle O'Malley

**Affiliations:** 1 International Training and Education Center for Health, University of Washington, Seattle, Washington, United States of America; 2 Department of Global Health, University of Washington, Seattle, Washington, United States of America; 3 National Institute of Health, Mozambique Ministry of Health, Maputo, Mozambique; 4 Department of Human Resources, Mozambique Ministry of Health, Maputo, Mozambique; 5 International Training and Education Center for Health, Maputo, Mozambique; World Health Organization, Switzerland

## Abstract

**Introduction:**

Mozambique suffers from a critical shortage of healthcare workers. Mid-level healthcare workers, (Tecnicos de Medicina Geral (TMG)), in Mozambique require less money and time to train than physicians. From 2009–2010, the Mozambique Ministry of Health (MoH) and the International Training and Education Center for Health (I-TECH), University of Washington, Seattle, revised the TMG curriculum. To evaluate the effect of the curriculum revision, we used mixed methods to determine: 1) if TMGs meet the MoH's basic standards of clinical competency; and 2) do scores on measurements of clinical knowledge, physical exam, and clinical case scenarios differ by curriculum?

**Methods:**

T-tests of differences in means examined differences in continuous score variables between curriculum groups. Univariate and multivariate linear regression models assess curriculum-related and demographic factors associated with assessment scores on each of the three evaluation methods at the p<0.05 level. Qualitative interviews and focus groups inform interpretation.

**Results:**

We found no significant differences in sex, marital status and age between the 112 and 189 TMGs in initial and revised curriculum, respectively. Mean scores at graduation of initial curriculum TMGs were 56.7%, 63.5%, and 49.1% on the clinical cases, knowledge test, and physical exam, respectively. Scores did not differ significantly from TMGs in the revised curriculum. Results from linear regression models find that training institute was the most significant predictor of TMG scores on both the clinical cases and physical exam.

**Conclusion:**

TMGs trained in either curriculum may be inadequately prepared to provide quality care. Curriculum changes are a necessary, but insufficient, part of improving TMG knowledge and skills overall. A more comprehensive, multi-level approach to improving TMG training that includes post-graduation mentoring, strengthening the pre-service internship training, and greater resources for training institute faculty may result in improvements in TMG capacity and patient care over time.

## Introduction

The introduction and rapid scale up of Mozambique's Tecnico de Medicina Geral (TMGs) healthcare cadre, analogous to Clinical Officers in other settings, was driven by a persistent shortage and uneven distribution of medical doctors (MDs). In 2007, approximately 550 doctors served Mozambique's population of over 18 million, a ratio of 3 physicians per 100,000 people [1]. TMGs (who are required to have three years of secondary school and pass a national selection exam before formal training), are expected by the Ministry of Health (MoH) to function as mid-level health care providers and to perform complex clinical tasks in the areas of general medicine, pediatrics, obstetrics, emergency services and minor surgery. Given the less costly and shorter training period for TMGs compared to MDs (30 months vs. six to seven years), TMGs may fill a critical niche in the national health system, especially in rural health settings where few doctors practice. However, the growing burden of HIV/AIDS and other infectious diseases over the last decade challenges the skills of the TMG [2], many of whom now must regularly diagnose and treat complicated AIDS-related cases while relying on insufficient laboratory, pharmacy and referral systems that are common in locations where TMGs practice.

The need to generate more TMGs and to provide them with better professional training is a priority for the Mozambique MoH. Therefore, from 2009–2010, the Training Directorate of the MoH with the assistance of the International Training and Education Center for Health (I-TECH), University of Washington, Seattle, completely overhauled the TMG curriculum. The overarching goal of the curriculum revision was to strengthen the clinical competencies of these mid-level clinicians with the ultimate goal of improving healthcare for the people of Mozambique. The process of developing the new curriculum included a review and revision of the TMG Terms of Reference (including skill and knowledge competencies) to more closely align with the epidemiological profile of the country; the resulting curriculum increased emphasis on malaria, malnutrition, HIV, and TB. The collaborative effort also resulted in a change from the initial curriculum that was subject- and classroom-based to a revised curriculum organized around body systems that places increased emphasis on hands-on practice and mastery of clinical skills. Health Training Institute (HTI) faculty was involved in reviewing initial drafts of the curriculum, and their feedback was incorporated into the revision process. Faculty development and implementation of the revised curriculum began in July 2010 including a multiple-day training of faculty and administrators to help ensure consistent and confident implementation of the revised curriculum at each HTI.

All training, initial and revised, was implemented at the HTIs of the MoH. Although HTIs vary in size and scope, all HTIs are located in urban or peri-urban centers in the provinces with campuses that include administration buildings, classrooms, practice laboratories, library and housing facilities. HTIs educate all health professionals other than MDs, including nurses, health agents, and non-medical specialties such as laboratory technicians. In either nationally-standardized curriculum, a mixture of full, part-time, and adjunct faculty implement courses at each HTI. The final class graduating under the initial curriculum was December, 2012. All eight HTIs that educate TMGs across the country now employ only the revised curriculum. Additional details on the processes of curriculum development and program implementation is detailed in a separate paper [3].

To evaluate the effect of the curriculum revision on the skills and capacities of the TMGs, we use mixed methods to determine: 1) to what extent did TMG graduates meet the MoH's basic standards of patient care, measured by their clinical competency? and 2) did cohorts trained in the revised curriculum score higher on measurements of clinical knowledge, physical exam procedures and solving clinical case scenarios, than those trained in the initial curricula? We compare clinical decision-making, clinical knowledge, and ability to perform a physical exam among 112 TMGs trained under the initial curriculum to 189 TMGs trained under the revised curriculum at two measurement periods: 1) immediately prior to graduation; and 2) ten months after graduation. For both curriculum groups at graduation only, qualitative interviews were conducted with key informants from the training institutions and with focus groups of TMGs to gage their perception of the strengths and weaknesses of their curriculum and to better understand factors that facilitate or hinder clinical competency. The results presented here use data from TMGs at baseline, immediately before graduation.

It is expected that the results of the study will inform the MoH in the development of practical, relevant ways to improve the quality of pre-service training for non-physician health cadres in Mozambique and could be used to inform policies and decisions regarding pre-service education for all mid-level health workers. If shown to be effective, this curriculum design could be applicable to pre-service curricula for other health worker cadres in Mozambique and in the region.

## Materials and Methods

### Ethics Statement

The study was approved by both the Centers for Disease Control in Atlanta, Georgia, USA and Mozambique's National Committee for Health Bioethics (protocol #5741/002, July 27, 2011). The internal review board of the University of Washington determined that this evaluation was not human subjects research but a routine program evaluation, granting a non-research determination. A part of the approved protocol, a comprehensive study consent form was read to all potential TMG participants, and participation was voluntary. Students could complete their training without participating in the study. Each voluntary participant signed the informed consent for all quantitative and qualitative measurements prior to participation. Consent documentation was retained by the INS and stored in a locked cabinet.

### Study Design

**Figure 1:**
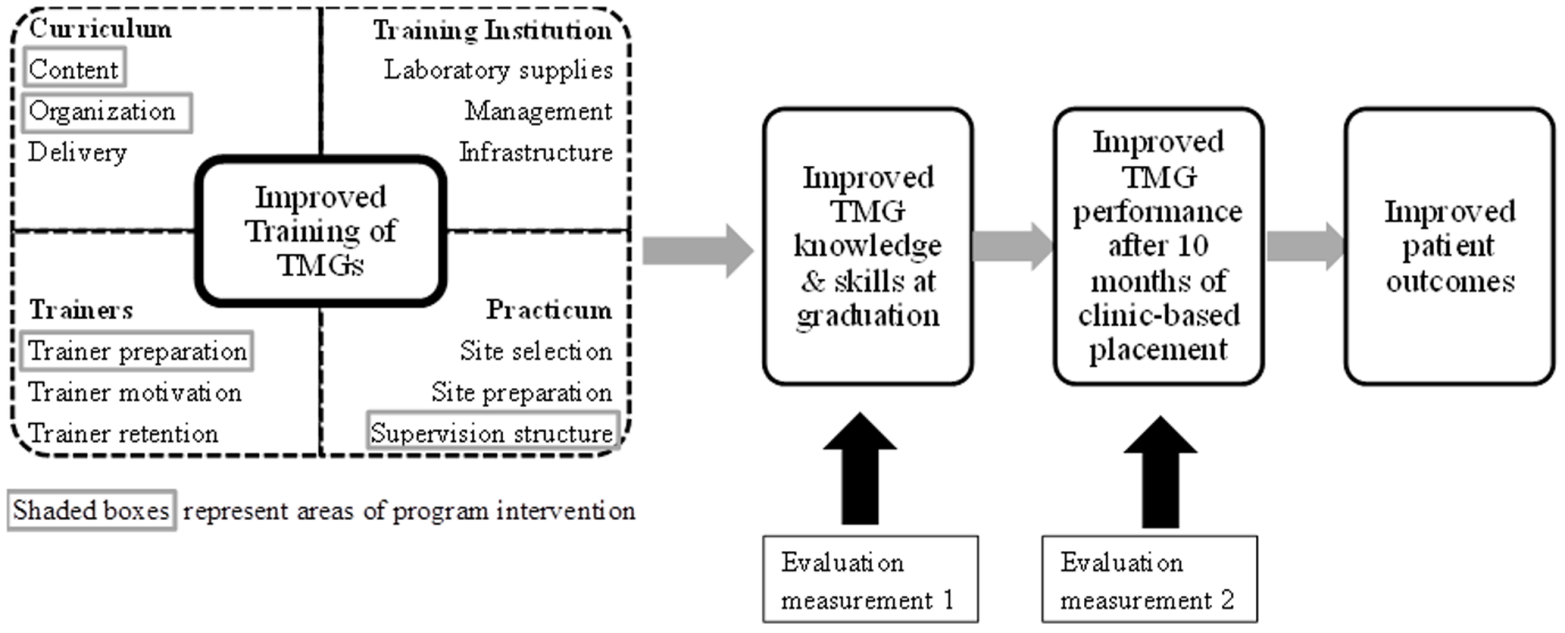
Program logic model for intended intervention effects of curriculum intervention on TMG performance.

This post-test only comparison group evaluation will compare clinical knowledge and reasoning, ability to perform a physical exam, as well as the degree to which specific standards of clinical care are met by 112 TMGs trained under the initial curriculum with 189 TMGs trained under the revised curriculum at two measurement periods: immediately prior to graduation; and after ten months of clinical experience. The study design, outlined in Figure 1, was informed by previous research on evaluation of healthcare worker training [4]. In Figure 1, boxes depict the areas where the intervention intended to affect training quality– including curriculum revision and the complementary trainer preparation and practicum support that accompanied implementation. The evaluation sought to measure the overall effects of the training on TMG knowledge and skills at two time points. This paper presents the results from both groups at graduation only.

### Study population

TMG students eligible to graduate from all Health Training Institutes (HTIs) between September 2011 and July 2013 were recruited for voluntary enrollment in the study. Measurement at graduation took place in December 2011 in four HTIs that implemented the initial curriculum. Three HTIs graduated students in the revised curriculum in 2012, and an additional four HTIs graduated revised TMGs in July 2013. TMG students only took courses in their specific curriculum, minimizing opportunities for contamination.

### Sample size

The sample size was calculated based on an expected 18% difference in mean scores between the initial and revised curriculum groups, increasing from a hypothesized 75% to 88.5%. Using G*power sample size calculator [5], a total sample size of 280 across 2 groups with 2 measurements for each participant, we have 80% power to detect an effect size of 0.18.

### Assessment methods

#### Qualitative

Facilitators and barriers to clinical competency were gathered through semi-structured interviews and focus groups following the clinical competency assessment at graduation. Interviews were conducted with HTI administrators or faculty; focus groups were conducted with 8–16 graduating TMGs per HTI. Weaknesses in the quality of the qualitative data identified in the first two rounds of data collection led to changes in the focus group guide for the final round of data collection for the revised curriculum group. Key informant and focus group questions are listed in Table 1.

**Table 1:** Key informant and focus group questions.

2011 and 2012 data collection (initial and revised)		2013 data collection (revised only)	
HTI Key informant interviews:	TMG Focus groups:	HTI Key informant interviews:	TMG Focus groups:
1. How are TMG skills defined and measured under the initial/revised? Can you describe how the TMGs acquire and practice skills?	1. Where do you think your knowledge and skills are particularly strong?	1. Describe the strengths and weaknesses of the training process for the revised curriculum.	1. Describe the strengths and weaknesses of your (TMG) training
2. What are the strengths of the TMG training under the initial/revised?	2. What clinical skills are the most difficult for you? What are the barriers to mastering these skills?	2. In terms of the lecture-based training components: What were the strengths and weaknesses of the lectures?	2. In terms of the lecture-based training components: What were the strengths and weaknesses of the lectures?
3. What are the weaknesses of the TMG training under the initial/revised? What are the barriers to TMGs learning these skills?	3. What are the strengths and weaknesses of your TMG curriculum?	3. In terms of the practical training components (labs), what were the strengths and weaknesses of the practice components?	3. In terms of the practical training components (labs), what were the strengths and weaknesses of the practice components?
4. Are there are specific skills that should be emphasized in the TMG training?	4. What specific skills should be emphasized in TMG training?	4. Internships: What were the constraints faced at the Health Centers and how could they be overcome?	4. Internships: What were the strengths and weaknesses of your internship experience?
5. What are the differences in terms of TMG learning between the initial curriculum and the revised curriculum?	5. How could your training be strengthened to better prepare you for your work?	5. What kind of additional support do you think the institution needs to improve TMG training?	5. In what technical/clinical areas do you feel prepared and in what areas do you feel weak? Why?
			6. What do you think can be done to improve TMG training?

#### Quantitative

To improve the assessment and overcome flaws in any one evaluation method [6] the evaluation of TMGs assessed clinical competency through three methods: 1) case scenarios to assess performance of clinical standards and clinical reasoning; 2) a written exam using both multiple-choice and case-study format to assess clinical knowledge; and 3) a mock physical exam using a standardized checklist and a paid, healthy, physical examination subject. The clinical skills and knowledge assessed through these multiple methods was reviewed to reflect the expected TMG performance as established in the MoH-defined competencies for TMGs. All assessments were piloted by the INS and ITECH with TMGs not involved in the study; assessments were subsequently revised.

Clinical simulation through case scenarios (vignettes) was chosen as a principle mean to obtain standardized, objective measurements of both clinical competence and reasoning skills of TMGs [7]. While real-life observation in clinic settings might be advantageous, possible variation in the types of clinical consultations observed, the need to control for variables such as the availability of supplies and diagnostic tools, and concerns about both timing and funding required for observation led to the use of clinical case scenarios. The steps to creating case scenarios included a review of the scope of practice and expected clinical competencies of TMGs, agreement between stakeholders on the most common diseases and diagnoses encountered by TMGs in routine practice, categorization of the required competencies, creation of structured case simulations of an appropriate level by clinical consultants, piloting with TMGs, and revision based on feedback. Final clinical scenarios were developed based on MoH guidelines and clinical standards for five common groups of conditions in Mozambique: suspicion/management of HIV infection; respiratory infections including TB; diarrheal illness; suspicion/treatment of malaria; and emergencies (including obstetric and pediatric emergencies). Twenty-five case studies were developed with the aim of five equivalent scenarios per topic group. Clinical evaluators administered case scenarios orally and recorded answers on a standardized scoring sheet. Trainings carried out for clinical evaluators in administration of the tools and standardized scoring across all case scenarios helped ensure that TMGs of equal levels would score similarly on the same case when observed by different evaluators. TMGs were randomized to one clinical scenario within each of the five topical groupings for a total of five case scenarios per data collection round. Case scenarios were implemented for each TMG individually, with the 5 cases taking approximately1-2 hours.

In addition to case simulation, a multiple-choice test was implemented to test a broader range of knowledge [8] and provide an additional assessment well recognized as a reliable and valid as a measure of clinical competence [9]. A 100 question test was developed and divided into 20 topics with 5 questions per topic area. Thirty unique knowledge tests were developed by randomly selecting two of the five questions per topic area for a total of 40 questions per exam. This multiple exam method was implemented to reduce cheating between TMGs who would take the exam in a small classroom with adjoining tables. The knowledge exam was implemented in a group setting and allotted 60 minutes.

Lastly, physical examination skills were tested through a standardized checklist administered during a mock physical exam, excluding genital and rectal exams. The use of task-based methodology to assess skill demonstration and variation has been demonstrated in the context of both clinical performance and curriculum evaluation [10]. The physical exam was implemented with the assistance of a healthy individual who volunteered as a physical exam recipient, receiving a nominal fee for their time. Each physical exam was conducted individually in standardized clinic rooms and limited to 15 minutes.

### Outcome measures

Outcome variables include: 1) continuous variable of average score across the 5 clinical case scenarios used to assess competency in specific clinical standards; 2) continuous score on the written, multiple choice exam to assess clinical knowledge and reasoning leading to decision-making on patient care for various conditions common to resource-poor countries; and 3) continuous score on the checklist used to assess physical exam skills.

Independent variables include: 1) curriculum type (initial vs. revised); 2) training institution; 3) demographic variables including age, marital status and gender.

### Evaluation team

To reduce possible bias in the evaluation, I-TECH subcontracted with Mozambique's National Institute of Health (Instituto Nacional de Saúde, INS), an autonomous technical and scientific institution subordinated to the Ministry of Health. INS responsibilities included collaboration on protocol development; assessment tool drafting and pre-testing; field team recruitment; quantitative and qualitative data collection; field logistics; data entry; data transcription, and first-line data quality assurance. I-TECH responsibilities included data quality assurance, data coding, and data analysis. A senior researcher from I-TECH and a senior clinician from INS were responsible for training the clinical evaluators who conducted the quantitative evaluations. MoH retains ownership of data.

### Team training and field implementation

The INS led a five-day training prior to each implementation round that included study ethics, study design overview, preparation of field materials, field protocols, and evaluation task training. Medical doctors from provinces where HTIs are located were selected by the local health authority to serve as clinical evaluators and were rigorously trained on all assessment types prior to each study round. Two or three evaluators were selected to participate in the training and evaluation per HTI site. Practice evaluations and inter-rater exercises were performed during the five day training to reach standardization among the evaluators. Study coordinators reviewed logistic preparation and assisted with the training. At graduation, an INS team comprised of a coordinator, study assistant, and 2–3 evaluators was dispatched to each HTI. INS teams in each location explained the study, administered the written informed consent, filled demographic information, and solicited both immediate and follow-up contact information. Participants were assigned a study number for all evaluation materials to reduce potential for bias or data tampering. Names and study numbers were not linked during field implementation or analysis. A staff member from I-TECH observed the first week of field implementation.

### Data Analysis

All data was collected, entered, and analyzed in Portuguese. Quantitative data from the clinical case scenarios, physical exam, and knowledge tests were analyzed using STATA 11.0 [11]. T-tests of differences in means were used to examine differences in continuous score variables between curriculum groups. Univariate linear regression models determined whether key factors of interest were significantly associated with assessment scores on each of the three evaluation methods. Factors significant at the p<0.05 level in univariate models were included in multivariate models. Preliminary analysis of case scenario data detected significant differences in mean scores within topical areas for both curriculum groups, demonstrating variation in the difficulty of the content (not shown). Therefore, analysis of differences in clinical case scores were adjusted by clinical case. Qualitative data was coded and thematically analyzed to complement quantitative analysis using Atlas.ti 5.0 [12].

## Results

### Quantitative

Demographics of 112 and 189 TMGs trained in the initial and revised curriculum, respectively, are presented in Table 2. No TMG refused participation in either round. The number and proportion of TMGs from each HTI are presented: four schools had students trained in both curricula (Chimoio, Beira, Quelimane, Pemba) while three additional schools (Tete, Nampula, Chicumbane) had students only from the revised. There are no significant differences in sex, marital status and age between curriculum groups. Of those TMGs trained in the initial curriculum, mean scores at graduation were 56.7%, 63.5%, and 49.1% on the clinical cases, knowledge test, and physical exam, respectively (Table 3). Mean scores for the revised curriculum TMGs at graduation (Table 3) were 57.3%, 62.6%, and 49.7% on the clinical cases, knowledge test, and physical exam, respectively. Revised curriculum scores did not significantly differ from those in the initial curriculum on any of the three assessment types.

**Table 2:** Comparison of demographic characteristics of TMGs in Initial vs. Revised curriculum*.

Characteristics	Initial (N = 112)	Revised (N = 189)	p-value
	# (%)	# (%)	
Sex			0.309
Male	77 (69.0)	119 (62.9)	
Female	35 (40.0)	70 (37.1)	
Marital status			0.524
Single	86 (77.0)	151 (79.9)	
Married	26 (23.0)	38 (20.11)	
Training institute			-
Chimoio	34 (30.4)	28 (14.8)	
Beira	27 (24.1)	30 (15.9)	
Quelimane	29 (25.9)	28 (14.8)	
Pemba	22 (19.6)	25 (13.2)	
Tete	-	24 (12.7)	
Nampula	-	24 (12.7)	
Chicumbane	-	30 (15.9)	
Age			0.310
Under 25 years	66 (58.9)	100 (52.9)	
25+	46 (40.1)	89 (47.1)	

*Results from chi-square tests of comparisons of means.

**Table 3:** Comparison of mean assessment scores* between TMGs in Initial vs. Revised curriculum at graduation.

	Initial (N = 112)	Revised (N = 189)	P = value
Clinical cases overall			0.70
Mean	56.7	57.3	
IQR	47.8–64.7	49.1–66.2	
SD	13.9	12.7	
Knowledge tests			0.54
Mean	63.5	62.6	
IQR	55.5–70.0	55.5–70.0	
SD	10.7	11.8	
Physical exam			0.74
Mean	49.1	49.7	
IQR	40.9–57.0	41.0–57.8	
SD	14.3	12.3	

*Results of t-test of continuous scores. IQR: Inter-quartile range.

Results from linear regression models of factors that influence continuous scores on all three assessment types are presented in Table 4. Although students were randomized to case number within each topic, significant differences in mean scores within each topic group (not shown) suggested cases of unequal difficulty; therefore, analysis was adjusted to include the specific clinical cases each TMG received. For clinical case scores, only HTI had a significant influence on score with higher scores from Chicumbane (5.93; CI: 0.97, 10.90) and lower scores from Quelimane (−12.02; CI: −16.10, −7.96), Pemba (−5.12; CI−9.41, 0.83), Tete (−8.79; CI: −14.16, −3.41), and Nampula (−15.58; CI: −20.93, −10.22) as compared to students at Chimoio. No other factor significantly influenced scores on clinical cases. For the knowledge test, men (4.47; CI: 1.79, 7.14) singles (5.21; CI: 2.10, 8.32) and younger students (6.38; CI: 3.89, 8.89) scored significantly higher than women, married, or older students, respectively. The positive association between both male and younger age on knowledge score remain similar and significant in multivariate analysis. Lastly, on the physical exam, single students scored higher (5.54; CI: 1.97, 9.12) than married students. Again, HTI significantly influenced exam score: students from Beira (11.58; CI: 7.28, 15.88), Quelimane (8.49; CI: 4.20, 12.79), Pemba (8.78; CI: 4.24, 13.31), and Chicumbane (12.46; CI: 7.24, 17.67) all scored higher than students at Chimoio while students from Nampula scored significantly lower (−6.28; CI: −11.91, −0.65). In the multivariate model, also Table 4, associations between HTI and physical exam score remained significant with overall slightly diminished strength.

**Table 4:** Univariate and multivariate factors affecting scores by assessment type.

Characteristics	Clinical cases^t^	Knowledge Test		Physical Exam	
	Univariate	Univariate	Multivariate	Univariate	Multivariate
Training					
Revised	0.33 (−2.71, 3.37)	−0.83 (−3.51–1.85)		0.53 (−2.54, 3.59)	
Sex					
Male	0.04 (−3.11, 3.19)	4.47*** (1.79, 7.14)	4.05*** (1.40, 6.70)	2.82 (−0.27, 5.92)	
Marital status					
Single	3.33 (−0.30, 6.97)	5.21*** (2.10, 8.32)	1.99 (−1.22, 5.21)	5.54** (1.97, 9.12)	3.90* (0.56, 7.23)
Health training institute (HTI)					
Chimoio	—	—		—	—
Beira	−0.76 (−4.83,3.31)	−0.10 (−4.21, 4.00)		11.58*** (7.28, 15.88)	11.52*** (7.26 15.79)
Quelimane	−12.02*** (−16.10, −7.96)	−2.12 (−6.27, 1.98)		8.49*** (4.20, 12.79)	8.50*** (4.23, 12.77)
Pemba	−5.12* (−9.41,0.83)	1.71 (−2.61, 6.04)		8.78*** (4.24, 13.31)	8.23*** 3.71, 12.75)
Tete	−8.79*** (−14.16, −3.41)	−1.09 (−6.47, 4.28)		4.42 (−1.20, 10.05)	4.51 (−1.07, 10.11)
Nampula	−15.58*** (−20.93, −10.22)	−4.84 (−10.21, 0.54)		−6.28* (−11.91, −0.65)	−5.54* (−11.17, 0.09)
Chicumbane	5.93* (0.97, 10.90)	−3.23 (−8.21, 1.73)		12.46*** (7.24, 17.67)	12.23*** (7.05, 17.40)
Age					
Under 25	2.31 (−0.69, 5.33)	6.38*** (3.89, 8.89)	5.93*** (3.36, 8.50)	2.21 (−0.76, 5.18)	

Results from linear regression tables. 95% CI in parenthesis.

*p≤.05.

**p≤0.01.

***p≤.0.001.

t Adjusted for case.

### Qualitative

The same key informant interview and focus group guides were used for the initial curriculum group and for the first round of data collection among the revised group. Guides were changed for the second group of students under the revised curriculum group to better explore themes exposed by the initial quantitative findings. Among the initial curriculum school, in 2011, seven key informants and four focus groups were conducted; eight key informant and six focus groups were conducted in 2012; and additional twelve interviews and seven focus groups were conducted with the revised group in 2013. There were forty-five interviews and focus groups, combined. Of themes reported in more than half of all combined discussions, administrators and students noted three major weaknesses in the internship component of the training: the internship was too short; the tutoring received during the internship was of poor quality; and, overcrowding at the internship sites limited practice opportunities. Comments on how the rapid pace of the revised curriculum was detrimental for students or faculty was commonly noted, and discussions of weaknesses among HTI faculty and lack of hands-on practice time at the HTI often followed. On the positive side, both students and administrators frequently noted their appreciation of uniformity of the new curriculum within all HTIs, the provision of the course packets, and the organization of the training materials. However, both administrators and students complained that the number of course packets provided were insufficient, leading students to share learning materials between several students.

## Discussion

Although the revised curriculum was intended to improve TMG training and lead to improvements in both knowledge and practice in this low resource setting, the evaluation results found no difference between initial and revised curriculum groups across evaluation types: case studies, knowledge test, or physical examination. We also found that overall scores of TMGs trained by both the initial and revised curriculum were not as high as we initially expected in any assessment type. On average, TMGs of both groups scored below 50% on the physical exam, and scored only marginally higher on the clinical case scenarios with an average score of 57%. Students fared better on the knowledge exam, with an average score of 63%. Although some students did exceptionally well on one or more of the assessment types, few TMGs in the initial or revised curriculum scored near the expected 75% or 88% achievement, respectively, hypothesized at study onset. These results suggest that TMGs from either curriculum may not have sufficient training to provide high quality clinical services upon graduation and that the revision of the pre-service curriculum, alone, did not improve TMG knowledge or skills. Qualitative results help illuminate these findings and shed light on some of the potential causes for the lack of change of between curriculum groups.

Primarily, although the curriculum revision was intended to be implemented in the same way in all schools, significant HTI-based differences in clinical case and physical exam scores suggest that the facility-based differences may affect student achievement more than course content. Post hoc analysis of school-based differences (Table 5) further suggests that school-level characteristics influence TMG training over individual attributes and curriculum group. Among the revised curriculum group, average scores on the clinical cases significantly varied from a low of 45.9% in Nampula to a high of 67.3% in Chicumbane (p<0.000) while on the physical exam, Nampula again faired poorest with mean score of 37.0%, almost 20 points behind Pemba with an average score of 56.1 (p<0.000). Several possible factors may explain inter-HTI differences. First, both standardized curricula included national competency expectations; however, the initial curriculum relied on the HTI faculty to develop course content whereas the revised curriculum was supported by lecture-by-lecture content, timelines, and key messages, leaving little to be developed in-house by faculty. Therefore, it was expected that all TMGs under the revised curriculum would receive, and benefit similarly from, the same standardized course across HTIs. However, HTI-specific differences in financial resources, faculty skill, administrative motivation, and institute infrastructure were not standardized between schools and may result in implementation variation. It is of note that scores on the knowledge test, a more common form of assessment in Mozambique, showed no significant inter-HTI variation. It is possible that the knowledge test better reflected the training methods with which HTI faculty were more comfortable while the physical exam and clinical cases reflected a more integrated form of TMG learning and capacity – a skill that only some institutions and faculty were able to inculcate in their students. These inter-HTI differences suggest that changes in the curriculum alone, without additional changes at training institutes, will not be sufficient to raise TMG knowledge and practice.

**Table 5:** Results* from comparison of means (average scores) between schools by curriculum group.

Health Training Institute	Case Scenarios	P	Knowledge Test	P	Physical Exam	P
Initial (N = 112):		0.000		0.537		0.000
Chimoio	65.2		62.4		38.2	
Beira	56.4		61.8		57.1	
Quelimane	49.9		64.6		55.6	
Pemba	52.5		65.6		47.4	
Revised (N = 189):		0.000		0.077		0.000
Chimoio	57.0		65.7		49.3	
Beira	64.2		65.6		52.6	
Quelimane	49.4		58.8		47.7	
Pemba	60.6		65.6		56.1	
Tete	53.4		62.8		47.7	
Nampula	45.9		59.1		37.0	
Chicumbane	67.3		60.7		55.7	

*Results from F tests of ANOVA models of comparison of means.

In further exploration of HTI-based differences, the qualitative interviews reveal several key constraints which may have affected curriculum implementation. First, in order to meet national TMG training targets and more quickly address critical shortages in the healthcare workforce, the TMG training schedule was reduced from 36 months for the initial curriculum to 30 months for the revised program while maintaining the same competency expectations. Both administrators and student note that the quick pace of the revised curriculum puts teachers and students at a disadvantage. Administrators mentioned that some teachers have a hard time getting through the courses on the intended schedule while other faculty have little flexibility to make up classes if they were absent, leaving students with gaps in classroom or practice-based learning. For students, some TMGs may be unprepared for rigor of the new course having entered with poor secondary school preparation and insufficient study skills. Students and administrators both noted that the expectation of studying or teaching on nights, weekends, and holidays was detrimental to their ability to help students synthesize and retain information. Moreover, although many administrators and students spoke positively about the initial provision of course materials for all teachers and students by I-TECH, later semesters required that the HTI copy and provide these materials for their students using MoH funds. As most HTIs and students have few resources, five or six students often shared one course packet, severely curtailing learning. Furthermore, both administrators and students noted deficits in teacher quality and quantity including that the number of dedicated HTI teachers are few, many are tasked with training multiple cadres, some teachers do not have course-specific or appropriate levels of expertise, and existing expert teachers may be called away for other duties leaving vacancies. Lastly, although both administrators and students approved of the increased emphasis on practical versus classroom learning in the revised curriculum, many noted that there were too many students and too little time for the limited practice lab space at the HTI and that the laboratories, themselves, often lacked the basic materials such as stethoscopes or functional mannequins to practice.

Outside of the HTI environment, factors associated with the quality of the internship component of the training present clear challenges to TMG skill acquisition. As part of both curricula, TMGs complete several standardized internships of various lengths at local health facilities or hospitals. However, administrators and students in both curriculum groups agree that there were several critical weaknesses of this training component. First, the internship sites are currently pre-selected by the MoH and are located in major population centers in the same province as the associated HTI. Students in TMG, nursing, and medicine programs often share practice sites with a limited number of on-site tutors and patients from whom to learn and practice. The overcrowding at practice sites strains tutor availability to teach and student ability to practice key skills. Second, both students and administrators noted the limited time that students spend at internship sites, both in hours per day and in days or weeks per topic rotation. Students are often expected to complete internship activities in the morning and return to the HTI for discussion in the afternoon, a scheduling decision that further limits TMG flexibility to observe, practice, and perfect the knowledge and skills needed to correctly manage patients from diagnosis through treatment. Third, the poor quality of the internship tutoring was noted commonly by students and administrators. Students suggested that tutor absences, limited teaching time, overcrowding at practice sites, and tutor reluctance to teach according to TMG-required competencies decreased the value of their internships. Administrators pointed out problems with the arrival of MoH-provided incentives for tutors as an explanation for why some medical doctors, clinical officers, and nurses tasked with mentoring TMG students may lack the motivation or desire to train TMGs. Overall, these gaps in experiential learning may leave some TMGs poorly prepared to assume their duties after graduation, reducing the quality of patient care.

## Limitations

There are several limitations that affected the evaluation implementation and application of the results for policy or practice. Several deviations from the field implementation protocol may compromise the quality of the findings. First, the treatment section of the case studies (one of 5 sections) were updated between initial curriculum and revised curriculum to reflect guideline changes; therefore study instruments were not identical between curriculum groups. However, analysis of differences in scores (not shown) between groups on this section, alone, showed no significant variation and no further adjustment was made. Some challenges in the quality of study implementation at the field sites resulted in deviations from the initial randomization of students. As noted previously, the level of clinical cases varied significantly within topic groups. Although randomization meant that each student had an equal likelihood of getting a hard or easy case, the variation may have put some TMGs at a disadvantage. Second, the materials available to the TMGs to conduct the mock physical exam differed between and within schools (for example: some rooms had scales while others used only paper to represent a scale). This may have caused bias in the scores of TMGs, but we are unable to determine the direction. Moreover, the evaluation was not designed to examine inter-HTI differences quantitatively. Further research that explores differences among the training institutes might better inform an HTI-specific, tailored intervention. Lastly, it is possible that the lack of effect between curriculum groups may be explained by Type III error, or failure to implement the program as intended. Examples from process evaluations conducted as part of the implementation and reviewed as part of background research suggest that components of the curriculum and its intended support structures were not implemented. Without the specified course materials, hands-on training practice, and trained faculty required by the curriculum, it is possible that students did not receive the full benefits of implementation. Despite these limitations, we believe that the findings from this field study provide insights on the realities of implementing, and evaluating, a revision of healthcare worker pre-service training in a low resource setting.

This evaluation has several strengths. Primarily, to the best of our knowledge, this is the first standardized evaluation of this cadre of healthcare worker training in Mozambique. Also, the process of developing and implementing the evaluation was conducted in partnership with, instead of for, the MoH, which may help translate the findings more rapidly into policy changes at the national or HTI level. Moreover, the three-part assessment utilized for the evaluation provided a mechanism to triangulate responses, affording a more robust measure of performance. Additionally, the implementation of the study helped increase local capacity, improving the skills of the INS research team to conduct rigorous assessments of healthcare worker competency. Lastly, there is often a bias in the literature towards positive results. We believe that sharing the results of this study widely will help others learn from the possible weaknesses in the implementation of the curriculum revision program, contribute positively to discussions on how to improve health workers training, and help others avoid similar pitfalls in the future. Sharing the limitations of the design of our evaluation may also help others further strengthen evaluation of health training programs in the future.

## Conclusion and Recommendations

These results suggest that TMGs may be inadequately prepared to provide quality care immediately after graduation and that curriculum changes are a necessary, but insufficient, part of improving TMG knowledge and skills overall. Weaknesses at the HTIs and in TMG internship opportunities compromise the training of the TMGs, limiting the potential effectiveness of the curriculum revision, alone. We present several recommendations for administrators, faculty, and TMGs that may be helpful in further strengthening the TMG training. First, at the national level, recent reviews of task shifting efforts [13], [14], including in Mozambique [15], [16], suggest that mid-level healthcare professionals such as TMGs may provide comparable care to medical doctors when they are supported by complementary training, supervision, and worker retention efforts after placement [17]. Therefore, health facility-based interventions that aim to improve the motivation and professionalism of existing staff [18], strengthen supervision structures [19], and increase agency among mid-level staff to identify and solve problems [20] may lead to longer-term, sustainable improvements in TMG capacity. Additionally, at the HTIs, additional resources must be secured to: 1) provide each TMG with an individual course packet; 2) stock laboratories with the required supplies for the practice labs; 3) support additional, full-time, expert faculty exclusively for TMGs; and 4) improve learning and library resources including internet access. Complementing these HTI-based interventions, the internship experience must be strengthened to help TMGs solidify their skills and gain confidence. Efforts should be made to qualify new sites for internship placements beyond the cities and provinces in which the students train to decrease overcrowding and provide more practice opportunities while lengthening the internship component would further provide more time to observe diverse patient profiles and solidify skills. Simultaneous efforts to improve tutor ownership and mentorship abilities would increase the benefits of the hands-on training. Lastly, increasing applicant quality, and presenting realistic expectations of study and learning schedules, may help improve the selection pool and performance of future TMGs. A more comprehensive, multi-level approach to improving TMG training may result in sustainable improvements in TMG capacity and, ultimately, patient care over time.
